# Iso-α-acids, Hop-Derived Bitter Components of Beer, Attenuate Age-Related Inflammation and Cognitive Decline

**DOI:** 10.3389/fnagi.2019.00016

**Published:** 2019-02-04

**Authors:** Yasuhisa Ano, Rena Ohya, Keiji Kondo, Hiroyuki Nakayama

**Affiliations:** ^1^Graduate School of Agricultural and Life Sciences, The University of Tokyo, Tokyo, Japan; ^2^Research Laboratories for Health Science & Food Technologies, Kirin Company Ltd, Yokohama, Japan

**Keywords:** aging, cognitive decline, hippocampus, inflammation, iso-α-acids, memory

## Abstract

With the aging population rapidly increasing worldwide, preventive measures and treatments for age-related cognitive decline and dementia are of utmost importance. We have previously demonstrated that the consumption of iso-α-acids (IAA), which are hop-derived bitter compounds in beer, prevents the formation of disease pathology in a transgenic mouse model of Alzheimer’s disease (AD). However, the effect of IAA consumption on age-related cognitive decline is unknown. In the present study, we examined the effect of long-term and short-term dietary consumption of IAA, on age-related memory impairments and inflammation in the hippocampus of aged mice. When compared with young mice, aged mice showed impairment in spatial working memory during the Y-maze spontaneous alternation test, impairment in object recognition memory during the novel object recognition test (NORT), a pro-inflammatory hippocampal microglial phenotype with increased CD86 expression and inflammatory cytokine production, increased levels of glutamate and amyloid β_1–42_, and decreased levels of dopamine (DA). In aged mice fed IAA for 3 months, the age-related alterations in memory, microglial inflammation, and glutamate, amyloid β_1–42_, and DA levels were all significantly attenuated. Additionally, the oral administration of IAA for 7 days in aged mice with memory impairment, also improved spatial and object recognition memory. These results suggest that IAA consumption prevents inflammation in the hippocampus and ameliorates age-related cognitive decline.

## Introduction

The increasing burden of dementia and cognitive impairment in rapidly expanding aging populations is shouldered not only by patients and their families but also by national healthcare systems. The lack of an effective disease-modifying therapy for dementia has garnered increasing attention on preventive approaches, such as diet, exercise, and learning. Etiological studies on lifestyle suggest that low to moderate consumption of alcohol, such as wine and beer, may reduce the risk of cognitive decline and the development of dementia. Individuals who consumed low to moderate levels of alcoholic beverages on a daily basis showed a significantly lower risk of developing a neurodegenerative disease than those who either abstained from alcoholic beverages or drank heavily (Matsui et al., 2011; Neafsey and Collins, 2011; Horvat et al., 2015). These risk-reducing effects are thought to be because of the profile of compounds found in alcoholic beverages. Red wine is known to contain resveratrol, a polyphenolic compound with neuroprotective properties (Neafsey and Collins, 2011; Porquet et al., 2014; Witte et al., 2014). Conversely, beer has remained the most-consumed alcoholic beverage in the world for more than a thousand years; to date there are few reports on which constituents of beer could be beneficial for preventing cognitive decline.

Hops, the female inflorescences of the hop plant (*Humulus lupulus L*.), have been used in beer brewing since 822 AD and are used as both a preservative and a flavoring agent in the beer-brewing process. The bitter taste of beer originates from the α-acids found in hops. Owing to the fact that iso-α-acids (IAA) activate the peroxisome proliferator-activated receptor-γ (PPAR-γ; Yajima et al., 2004), the IAA found in beer have antioxidant and anti-metabolic syndrome properties and are also reported to prevent diet-induced obesity in rodents and to improve hyperglycemia, a result which has been confirmed in humans (Obara et al., 2009). We previously demonstrated that the long-term intake of IAA prevented Alzheimer’s pathology in transgenic model mice (Ano et al., 2017). IAA suppressed microglial inflammation induced by the deposition of amyloid β (Aβ) in the brain and prevented cognitive decline. Additionally, IAA activated PPAR-γ and regulated microglial phagocytosis and inflammation. Our group has also demonstrated that IAA suppressed microglial inflammation in tauopathy mice and improved obesity-induced cognitive impairment by suppressing inflammation induced by a high-fat diet (Ano et al., 2018b; Ayabe et al., 2018). However, the effects of IAA on age-related cognitive decline and neuronal dysfunction have not yet been demonstrated. Most cases of cognitive decline are associated with aging; consequently, in this study, we examined the effects of IAA consumption on cognitive impairment and brain inflammatory processes in aged mice.

## Materials and Methods

### Preparation of Iso-α-acids (IAA)

The α-acids predominantly comprised of the following three congeners: cohumulone, humulone, and adhumulone. During the brewing process, they were each isomerized into two epimeric isomers: cis-IAA and trans-IAA. We used isomerized hop extract (IHE; Hopsteiner, Mainburg, Germany) containing 30.5% (w/v) IAA and 65% H_2_O as the IAA sample for our experiments. Based on the analysis in our previous report, IHE comprises six isomers: trans-isocohumulone (1.74% w/v), cis-isocohumulone (7.61% w/v), trans-isohumulone (3.05% w/v), cis-isohumulone (14.0% w/v), trans-isoadhumulone (0.737% w/v), and cis-isoadhumulone (3.37% w/v; Ano et al., 2017).

### Animals

Male C57BL/6J mice (Charles River Japan, Tokyo, Japan) were maintained at the Kirin Company Ltd. The Animal Experiment Committee of Kirin Company Limited approved all experiments, which were conducted between 2016 and 2017 in strict accordance with their guidelines. We made every possible effort to minimize suffering. Mice were fed a standard purified rodent diet (AIN-93M, Oriental Yeast, Tokyo, Japan).

Our previous study showed that dietary intake of 0.05% (w/w) IAA reduced inflammation in the brain of Alzheimer’s model mice (Ano et al., 2017). To study the effect of long-term IAA intake on age-related cognitive decline, aged mice (68 weeks of age) were fed AIN-93M with or without 0.05% (w/w) IAA for 3 months. Young control mice (7 weeks of age) were fed AIN-93M diet without IAA for 3 months. After a 3-month dietary intervention, the aged mice fed IAA (*n* = 10), control aged mice not fed IAA (*n* = 9), and control young mice (*n* = 12) underwent behavioral evaluation, after which brain samples were obtained for subsequent biochemical evaluation. The body weight of the aged mice with and without IAA did not differ between groups (Supplementary Figure S1A). To study the effect of short-term IAA intake, mice aged at 22 months were intragastrically administered IAA at either 0 mg/kg (distilled water as vehicle, *n* = 13) or 1 mg/kg (*n* = 14) for 9 days. Young mice aged at 7 months, that were administered with vehicle (*n* = 15), served as additional controls. At 7 days, mice were subjected to the spontaneous alternation test; at 8 and 9 days, mice were subjected to the novel object recognition test (NORT) at 1 h after the IAA administration.

### Spontaneous Alternation Test

We evaluated spatial memory by performing a spontaneous alternation test using a Y-maze in accordance with our previous study (Ano et al., 2018a). The Y-maze is a 3-arm maze with equal angles between each black polyvinyl plastic arm (25 cm long × 5 cm wide × 20 cm high). Each mouse was initially placed in the start arm of the maze, and the number and sequence of subsequent arm entries was recorded over 8 min. We defined the alternation score (%) for each mouse as the ratio of the actual number of alternations to the total possible number of alternations (defined as the total number of arm entries minus 2) multiplied by 100 as follows: alternation score (%) = [(number of alternations)/(total arm entries − 2)] × 100.

### NORT

We evaluated episodic memory by performing NORT in accordance with our previous study (Ano et al., 2018a). NORT was performed during the light period in a polyvinyl chloride box (25 cm × 40 cm × 20 cm) without a roof. For the acquisition trial, we used a pair of wooden triangle poles (4.5 cm × 4.5 cm × 4.5 cm) or wooden pyramids (4.5 cm × 4.5 cm × 4.5 cm); for the retention trial, we used one triangle pole or pyramid as the familiar object and a golf ball (4.5 cm diameter) as the novel object In all trials, we placed the objects 7.5 cm from the corner of the box. In the acquisition trial, each mouse was allowed 10-min exploration time in the box with the two identical objects at 1 h after intragastric administration of the test sample. At 24 h after the acquisition trial and 1 h after intragastric administration, mice were allowed to explore the box with the novel and familiar objects for 5 min. The discrimination index (DI) was calculated by dividing the difference in time taken to explore the novel object and the familiar object, by the total time spent exploring both objects, that is, DI = (novel object exploration time − familiar object exploration time)/(total exploration time). Using this method, equal exploration of both objects was indicated by a DI of 0.

### Measurements of Activity, Food Intake, and Water Intake in Home Cage

We monitored the amount of food intake, water intake, and ambulatory activity in the home cages using a three-point meter for 72 h (O’HARA & Co., Ltd., Tokyo, Japan). To monitor home cage activity, the interruption of infrared beams positioned on the X and Y axes around the cage detected the position of the mouse, automatically measuring their position and movement in their home cages. In this experiment, we measured the moving distance of each mouse over 5 min for a total of 72 h.

### Aβ and Cytokine Measurement

To measure levels of cytokines, Aβ, and Tau, we homogenized the hippocampus of the left hemisphere in TBS buffer (Wako) with a multi-beads shocker (Yasui Kikai, Osaka, Japan). After centrifugation at 50,000× *g* for 20 min, we collected the supernatant. We measured the total protein concentration of each supernatant with a BCA protein assay kit (ThermoScientific, Yokohama, Japan). We assayed the supernatant to quantify soluble Aβ_1–42_ (Wako), Tau (ThermoScientific), and phosphorylated Tau (pTau, pS199, ThermoScientific) by ELISA and cytokines by a Bio-Plex assay system (Bio-Rad, Hercules, CA, USA).

### Microglial Analysis

We isolated microglial cells from the mouse brain by magnetic cell sorting after conjugation with anti-CD11b antibodies, as described previously (Ano et al., 2015). Isolated CD11b-positive cells (>90% pure, as evaluated by flow cytometry) were cultured in DMEM/F-12 (Gibco, Carlsbad, CA, USA) supplemented with 10% fetal calf serum (Gibco, Carlsbad, CA, USA) and 100 U/ml penicillin/streptomycin (Sigma-Aldrich, St. Louis, MO, USA). We treated the microglia with a leukocyte activation cocktail containing GolgiPlus (BD Biosciences, San Jose, CA, USA) for 12 h, fixed and permeabilized them with a Cytofix/Cytoperm Fixation/Permeabilization Kit (BD Biosciences, San Jose, CA, USA), and stained the microglia with FITC-conjugated anti-mouse TNF-α (MP6-XT22, eBioscience, San Diego, CA, USA), APC/Cy7-conjugated anti-mouse CD11b (M1/70, BD Pharmingen), and APC-conjugated anti-mouse CD86 (GL1, eBioscience, San Diego, CA, USA) antibodies. We analyzed populations of cytokine-producing cells and the expression of cell markers with a flow cytometer (BD FACSCantoII, BD Biosciences, San Jose, CA, USA). We have expressed flow cytometry data as the mean median mode of the fluorescence intensity.

### Monoamine Analysis

To evaluate the levels of the monoamine dopamine (DA) and its metabolites in the brain, we homogenized tissue in 0.2 M perchloric acid (Wako) containing 100 μM disodium EDTA (Sigma-Aldrich, St. Louis, MO, USA). After centrifugation, we analyzed the supernatant using high-performance liquid chromatography (HPLC) with an EICOMPAK SC-5ODS column and a PREPAK column (Eicom, Kyoto, Japan) with electrochemical detection (ECD). The mobile phase comprised of 83% 0.1 M acetic acid in citric acid buffer (pH 3.5), 17% methanol (Wako), 190 mg/ml sodium 1-octanesulfonate (Wako), and 5 mg/ml disodium EDTA. For ECD, we applied a voltage of 750 mV against an Ag/AgCl reference electrode.

### Statistical Analyses

Data are presented as the mean with error bars representing the standard error of the mean (SE). We analyzed data by the one-way analysis of variance (ANOVA), followed by the Tukey-Kramer *post hoc* test. All statistical analyses were performed with Ekuseru-Toukei 2012 software (Social Survey Research Information, Tokyo, Japan). We considered a *p*-value of <0.05 to be statistically significant.

## Results

### Preventive Effects of IAA on Age-Related Memory Impairment in Aged Mice

**Figure 1:**
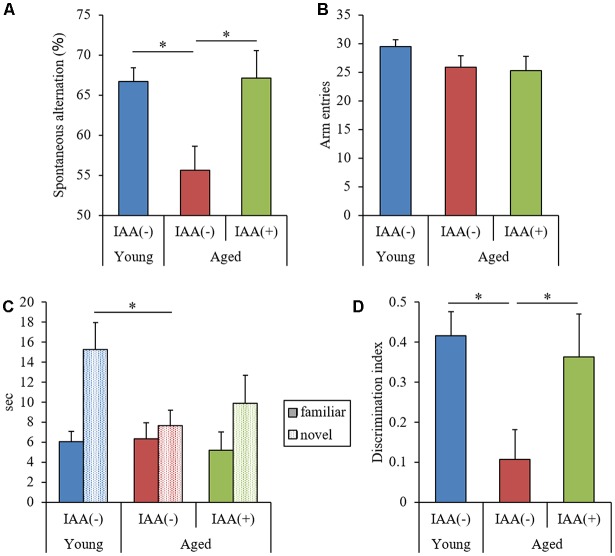
Behavioral evaluation of cognitive functions. We fed C57BL/6J mice aged 7 weeks (young) and 68 weeks (aged) diets containing 0% or 0.05% (w/w) Iso-α-acids (IAA) for 3 months. We then performed behavioral evaluation in young mice (*n* = 12) and aged mice with (*n* = 10) or without (*n* = 9) dietary IAA. **(A,B)** We evaluated spatial memory using a Y-maze spontaneous alternation test to measure spontaneous alternation score **(A)** and total arm entries **(B)**. **(C,D)** We evaluated object recognition memory using a novel object recognition test (NORT). We measured the time taken to approach a novel or familiar object **(C)** and the discrimination index (DI) in NORT **(D)**. Data are presented as mean ± standard error of the mean (SE). We calculated *p*-values shown in the graph by one-way analysis of variance (ANOVA), followed by the Tukey-Kramer test. *p < 0.05.

To evaluate the effects of IAA on memory impairment in aged mice, we fed mice a diet containing IAA for 3 months at 7 weeks old (young mice) and 68 weeks old (aged mice) and subjected them to behavioral memory evaluations using the spontaneous alternation test and NORT. In the Y-maze spontaneous alternation test, aged mice showed significantly lower alternation scores compared with young mice (Figure 1A), whereas the number of total arm entries was not different between the experimental groups (Figure 1B). In NORT, the time spent exploring the novel object (Figure 1C) and DI (Figure 1D) in aged mice were significantly decreased compared with those in young mice, whereas the total time taken to approach each object did not differ between groups. These results indicated that spatial working memory and object recognition memory declined with aging. However, IAA-fed aged mice showed significantly higher spontaneous alternations in the Y-maze (Figure 1A) and DI in NORT (Figure 1D) compared with the control aged mice, which indicated that dietary IAA intake for 3 months prevented age-related memory impairment. Food and water intake among the groups did not differ significantly (Supplementary Figures S1B,C, respectively).

### Preventive Effects of IAA on Inflammation in Aged Mice

**Figure 2:**
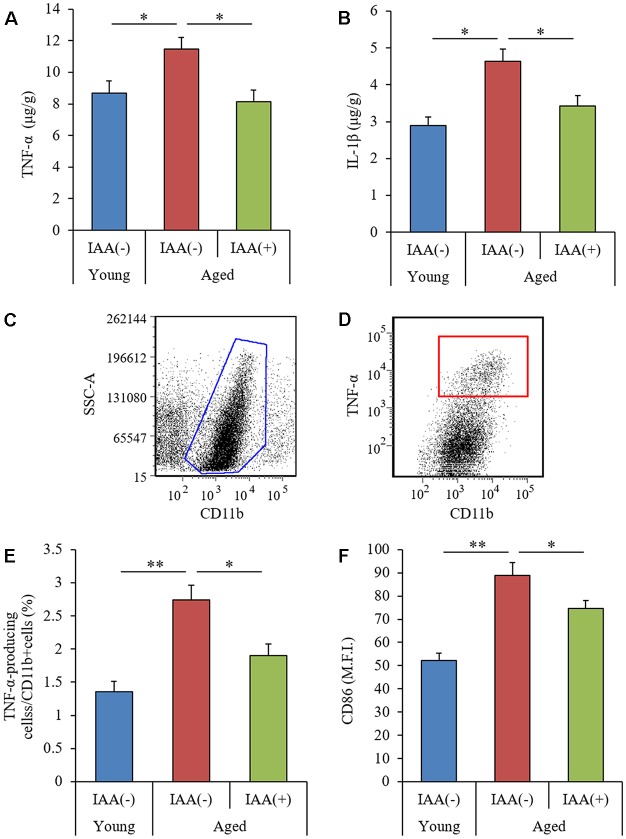
Characteristics of microglial inflammation in aged mice. We fed C57BL/6J mice aged 7 weeks (young) and 68 weeks (aged) diets containing 0% or 0.05% (w/w) IAA for 3 months. We then obtained brain tissue for the analysis of microglial inflammation from young mice (*n* = 12) and aged mice with (*n* = 10) or without (*n* = 9) dietary IAA. **(A,B)** The levels of TNF-α **(A)** and IL-1β **(B)** in the hippocampus. **(C,D)** Flow cytometry characterization of TNF-α-production in CD11b-positive microglia isolated with magnetic cell sorting. **(E,F)** We collected CD11b-positive microglia using magnetic cell sorting and analyzed them using a flow cytometer, examining the ratio of TNF-α-producing cells to CD11b-positive cells **(E)** and the expression of CD86 on CD11b-positive cells **(F)**. Data are presented as mean ± SE. We calculated* p*-values shown in the graph by one-way ANOVA, followed by the Tukey-Kramer test. *p < 0.05 and **p < 0.01.

To evaluate the effects of IAA on age-related inflammation in the brain, we measured the level of cytokines in the hippocampus and analyzed the phenotype of brain microglia. Aged mice showed significant increases in TNF-α and IL-1β in the hippocampus compared with young mice (Figures 2A,B, respectively). The percentage of CD11b-positive microglia producing TNF-α (as gated in Figures 2C,D) and the microglial expression of CD86 were significantly increased in aged mice compared with young mice (Figures 2E,F, respectively). These results suggested that age-related microglial-mediated inflammation was present in the brain. Long-term dietary IAA in aged mice was able to attenuate the age-induced increase in hippocampal TNF-α and IL-1β levels and microglial inflammation, indicating that IAA downregulated aged-related inflammation in the brain. These results indicate that aging induces inflammation in the mouse hippocampus and that this inflammation can be reduced by IAA consumption.

### Effects of IAA on Molecules Inducing Inflammation in Aged Mice

**Figure 3:**
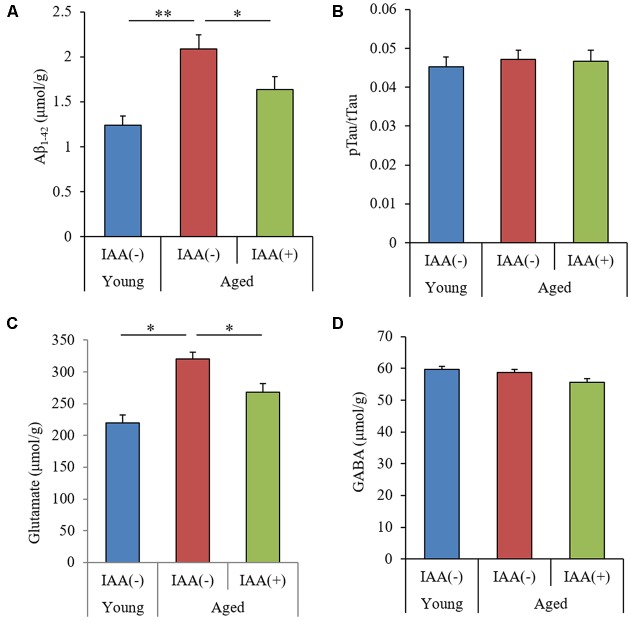
Levels of amyloid β (Aβ), Tau, and glutamate in the hippocampus of aged mice. We fed C57BL/6J mice aged 7 weeks (young) and 68 weeks (aged) diets containing 0% or 0.05% (w/w) IAA for 3 months and then obtained brain tissue for analysis from young mice (*n* = 12) and aged mice with (*n* = 10) or without (*n* = 9) dietary IAA. **(A,B)** The levels of Aβ_1–42_ **(A)** and phosphorylated Tau to total Tau (pTau/Tau; **B**) in TBS-soluble fractions of the hippocampus. **(C,D)** The amount of glutamate **(C)** and GABA **(D)** in the hippocampus. Data are presented as mean ± SE. We calculated the *p*-values shown in the graph by one-way ANOVA, followed by the Tukey-Kramer test. *p < 0.05 and **p < 0.01.

We measured the level of Aβ_1–42_, the ratio of pTau to total Tau (pTau/Tau), and the level of glutamate in aged mice to evaluate the effect of IAA on the production of inflammation-inducing molecules. Aβ is a major component of senile plaques (Hsiao et al., 1996), and the phosphorylation of Tau is known to cause neurofibrillary tangles (Wood et al., 1986). Glutamate can mediate neurotoxic effects and can induce inflammation in the brain (Novelli et al., 1988). We found that the hippocampus of aged mice had significantly higher levels of both TBS-soluble Aβ_1–42_ and glutamate compared with the hippocampus of young mice (Figures 3A,C, respectively), but the ratio of pTau/Tau and the level of the inhibitory neurotransmitter GABA in the hippocampus did not differ between groups (Figures 3B,D, respectively). The age-related increases in Aβ and glutamate levels were significantly attenuated in aged mice provided with dietary IAA. These results indicated that Aβ_1–42_ and glutamate, which have both been linked to inflammation and cognitive decline in the brain (Lambert et al., 1998; Brown and Bal-Price, 2003), increase in the hippocampus with aging and that dietary IAA was able to reduce the age-related increase in these molecules.

### Effects of IAA on Monoamine Production in Aged Mice

**Figure 4:**
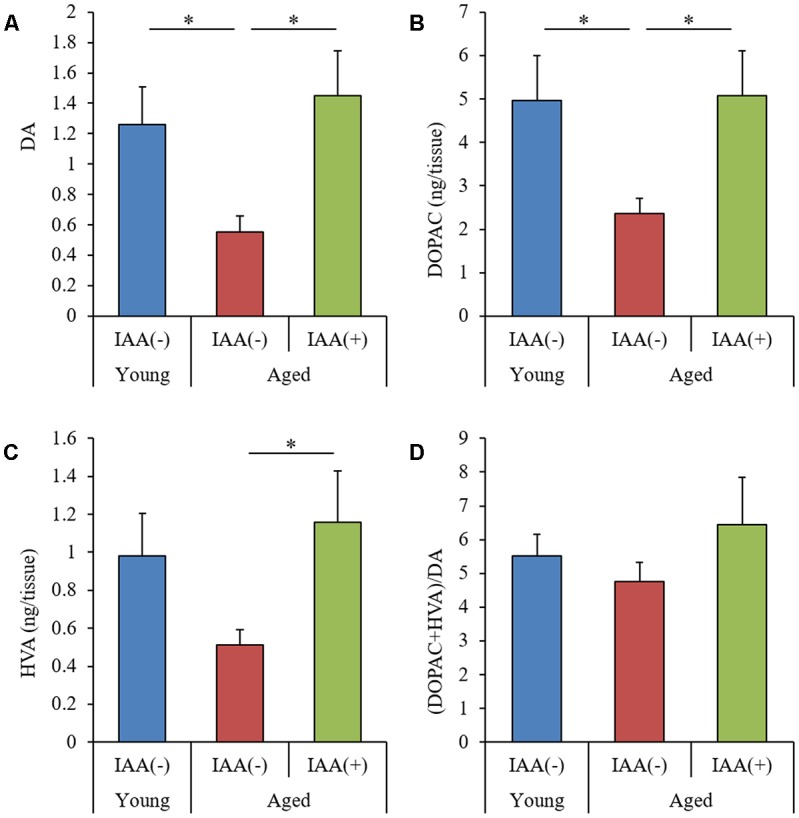
Levels of dopamine (DA) and its metabolites in aged mice. We fed C57BL/6J mice aged 7 weeks (young) and 68 weeks (aged) diets containing 0% or 0.05% (w/w) IAA for 3 months and then obtained brain tissue for analysis of monoamine content in young mice (*n* = 12) and aged mice with (*n* = 10) or without (*n* = 9) dietary IAA. **(A–C)** The amount of DA **(A)**, 3, 4-dihydroxyphenylacetic acid (DOPAC; **B**), and homovanillic acid (HVA; **C**) in the hippocampus. **(D)** The ratio of (DOPAC + HVA)/DA. Data are presented as mean ± SE. We calculated the *p*-values shown in the graph by one-way ANOVA, followed by the Tukey-Kramer test. *p < 0.05.

DA is thought to be crucial for hippocampus-dependent memory (Li et al., 2003; Chan et al., 2017). To evaluate the effects of aging and IAA on monoamine production, we used an HPLC-ECD system to measure the levels of DA and DA metabolites, 3,4-dihydroxyphenylacetic acid (DOPAC), and homovanillic acid (HVA) in the hippocampus. The levels of DA and DOPAC were significantly decreased in aged mice compared with young mice (Figures 4A,B, respectively). However, the level of HVA and ratio of (DOPAC + HVA)/DA were not significantly altered by aging (Figures 4C,D, respectively). The level of norepinephrine did not differ between the experimental groups. These results indicated that the dopaminergic system function declined with aging but that the age-related decreases in DA and DOPAC could be significantly attenuated in aged mice that are administered dietary IAA.

### Effects of Short-Term IAA Intake on Memory Impairments in Aged Mice

**Figure 5:**
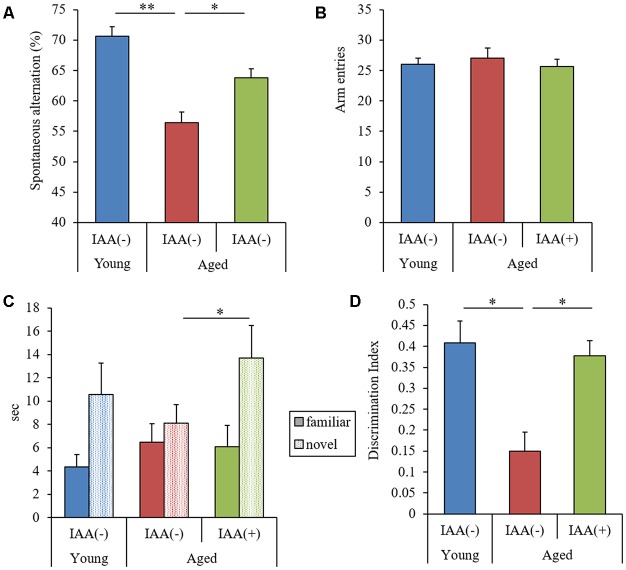
Effects of the short-term administration of IAA in aged mice. C57BL/6J mice aged 7 months (young) and 22 months (aged) were orally administered 0 or 1 mg/kg IAA for 9 days. We performed behavioral characterization using the spontaneous alternation test at day 7 of administration and the NORT at days 8 and 9 at 1 h after the oral administration. For these behavioral tests, we used young mice (*n* = 15) and aged mice with (*n* = 14) and without (*n* = 13) dietary IAA. **(A,B)** We measured spontaneous alternation score **(A)** and arm entries **(B)** in the Y-maze spontaneous alternation test to evaluate spatial memory. **(C,D**) To evaluate object recognition memory, we measured the time taken to approach a novel or familiar object **(C)** and DI in NORT **(D)**. Data are presented as mean ± SE. We calculated the *p*-values shown in the graph by one-way ANOVA, followed by the Tukey-Kramer test. *p < 0.05 and **p < 0.01.

We demonstrated that the long-term intake of IAA suppressed cognitive decline and inflammation in the brain of aged mice. Next, to evaluate the effects of the short-term intake of IAA on memory function, we administered aged mice (22 months of age) IAA at 1 mg/kg for 9 days. In this experiment, both the spontaneous alternation score in the Y-maze and DI in NORT were significantly lower in aged mice than in young mice (7 months of age; Figures 5A,D, respectively). The total number of arm entries in the Y-maze did not differ between groups (Figure 5B). However, aged mice that received short-term oral administration of IAA showed significant improvements in the Y-maze spontaneous alternation score, the time taken to approach a novel object, and DI compared with control aged mice without orally administered IAA (Figures 5A,C,D, respectively). These results indicated that the short-term intake of IAA improved spatial and object recognition memory impairment that occurred in aging.

## Discussion

Given the worldwide increase in aging populations, it is imperative to discover novel preventive and treatment therapies for cognitive decline. Our study demonstrated that the consumption of IAA reduced inflammation in the brain and prevented the cognitive impairment associated with normal aging in mice. Aged mice displayed microglial inflammation in the hippocampus and impairments in spatial working memory and object recognition memory; but both the inflammation and the cognitive impairments were reduced by long-term dietary administration of IAA. In addition to these long-term preventive effects, short-term IAA administration also improved age-related cognitive impairment in aged mice.

In aged mice, the Aβ level in the hippocampus was increased and occurred in conjunction with a microglial inflammatory response and cognitive decline. Aβ, a well-known agent of Alzheimer’s disease (AD; Kametani and Hasegawa, 2018), is gradually deposited in the brain over a long period, inducing chronic inflammation and accelerating disease pathology (Heppner et al., 2015). In aged mice, microglial inflammation is induced by Aβ and other antigens; activated microglia produce pro-inflammatory cytokines and reactive oxygen species, both of which lead to cognitive impairment (Sarlus and Heneka, 2017; Wendeln et al., 2018). Glutamate, a neurotransmitter that can confer neurotoxicity, is also produced by activated microglia (Barger et al., 2007; Takaki et al., 2012). Increased glutamate in the hippocampus of aged mice is thought to be involved in memory impairment (Tamminga et al., 2012). The short-term spatial memory tested in the Y-maze and long-term object recognition memory tested in NORT are hippocampus-dependent memory types (Cohen et al., 2013; Albani et al., 2014; Pioli et al., 2014). Inflammation in the hippocampus is reported to have impaired these types of memory, and the suppression of inflammation subsequently improved these memory impairments (Miwa et al., 2011; Abareshi et al., 2016). Evidence from these reports together with the findings in our present study suggest that dietary IAA is capable of suppressing microglial inflammatory responses in aged mice, which might be associated with the prevention of the decline in hippocampal memory associated with aging. The behavioral evaluations in the present study cannot discriminate which specific step in memory (acquisition, retention, or recall) was improved by IAA consumption. Further evaluation of hippocampus-dependent memory acquisition and retention using the Morris water maze or radial arm maze will further elucidate the effects of IAA on memory impairments with aging.

We have previously demonstrated, as a mechanism underlying the suppression of inflammation by IAA in aged mice, that IAA activates PPAR-γ (Yajima et al., 2004), increases the expression of the “Aβ receptor” CD36, promotes phagocytosis of Aβ (Yu and Ye, 2015), and promotes microglial activation toward the M2 anti-inflammatory type (Ano et al., 2017). In AD model mice, Aβ deposition and inflammation in the hippocampus were reduced by IAA administration (Ano et al., 2017). In the present study, the increased expression of CD86 and production of TNF-α in aging are characteristic of the M1 inflammatory type of microglia (Zhou et al., 2017). The administration of IAA reduced these characteristics, suggesting that IAA suppressed the activity of M1 microglia. These results suggest that IAA consumption shifts microglial activation toward an anti-inflammatory phenotype in aged mice by activating PPAR-γ. Some studies report that the long- and short-term administration of PPAR-γ agonists, such as pioglitazone and rosiglitazone, suppressed cognitive decline in aged transgenic mice (Heneka et al., 2005; Escribano et al., 2010), but to date, the effects of PPAR-γ agonists on normally aged mice have not been demonstrated. In the present study, we demonstrated for the first time that IAA, a PPAR-γ agonist, prevent age-related cognitive decline in naturally aged mice.

Our study also demonstrated that the levels of DA and its metabolite DOPAC in the hippocampus of aged mice were reduced compared with those of young mice, and this reduction was ameliorated in aged mice by IAA. Levels of DA and DOPAC in the nucleus accumbens of aged mice were reported to be reduced when compared with those of young mice, whereas those in the striatum did not differ between groups, but to date, there has been no report on DA levels in the hippocampus of aged mice (Winner et al., 2017). In the hippocampus, DA is crucial for both spatial memory and object recognition memory. Inflammation in the brain is also reported to reduce the production of DA (Coffeen et al., 2010); accordingly, lipopolysaccharide endotoxin treatment reduced DA and DOPAC levels in rats (Noworyta-Sokolowska et al., 2013). We suggest that inflammation in the aging brain induces the reduction of DA level in the hippocampus, resulting in memory impairment.

In conclusion, our study demonstrated that consumption of IAA, the hop-derived component that imparts a bitter taste to beer, suppresses microglial activation and attenuates aged-related memory impairment in aged mice. This finding supports those of existing epidemiological studies and our previous research. Various brain disorders, including dementia, depression, and chronic fatigue, are associated with inflammation in the brain. The consumption of IAA might support the treatment or even reversal of various inflammation-related conditions such as cognitive decline.

## Data Availability

The datasets generated for this study are available on request to the corresponding author.

## Author Contributions

YA conducted the experiment and wrote the manuscript. RO performed the experiment and the behavioral evaluations. KK and HN designed and conducted the research.

## Conflict of Interest Statement

YA, RO and KK are employed by Kirin Company Ltd. The remaining author declares that the research was conducted in the absence of any commercial or financial relationships that could be construed as a potential conflict of interest.

## Supplementary Material

FIGURE S1 Weight and food/water consumption in aged mice. We fed C57BL/6J mice aged 7 weeks (young; *n* = 12) and 68 weeks (aged) diets containing 0% or 0.05% (w/w) IAA for 3 months (aged with dietary IAA, *n* = 10; aged without dietary IAA, *n* = 9). **(A)** Body weight was monitored every month. **(B,C)** Food **(B)** and water **(C)** consumption over 72 h. Data are presented as mean ± SE. We calculated the *p*-values shown in the graph by one-way analysis of variance (ANOVA), followed by the Tukey-Kramer test.

## Abbreviations

Aβ: amyloid β
DA: dopamine
DOPAC: 3,4-dihydroxyphenylacetic acid
ECD: electrochemical detection
DI: discrimination index
HVA: homovanillic acid
HPLC: high-performance liquid chromatography
IAA: iso-α-acids
NORT: novel object recognition test
PPAR-γ: peroxisome proliferator-activated receptor-γ.
