# Supplementary figures and images for: Iso-α-acids, Hop-Derived Bitter Components of Beer, Attenuate Age-Related Inflammation and Cognitive Decline
Source: Front Aging Neurosci. 2019 Feb 4;11:16. doi: 10.3389/fnagi.2019.00016 (PMC6369178; doi:10.3389/fnagi.2019.00016)

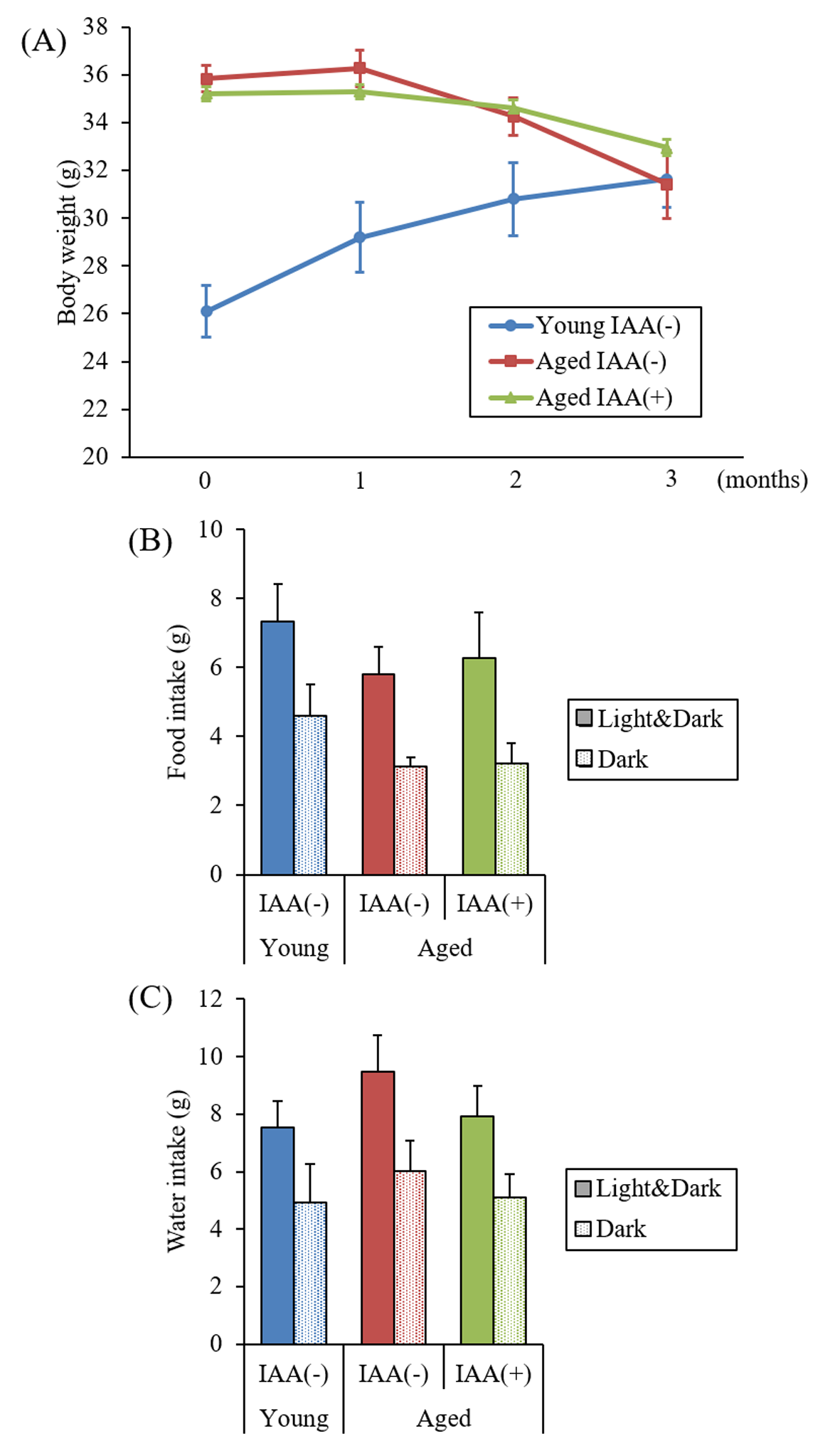

Supplement: FIGURE S1 — Weight and food/water consumption in aged mice. We fed C57BL/6J mice aged 7 weeks (young; n = 12) and 68 weeks (aged) diets containing 0% or 0.05% (w/w) IAA for 3 months (aged with dietary IAA, n = 10; aged without dietary IAA, n = 9). (A) Body weight was monitored every month. (B,C) Food (B) and water (C) consumption over 72 h. Data are presented as mean ± SE. We calculated the p-values shown in the graph by one-way analysis of variance (ANOVA), followed by the Tukey-Kramer test. [file Image_1.TIF]
